# Adult-Young Ratio, a Major Factor Regulating Social Behaviour of Young: A Horse Study

**DOI:** 10.1371/journal.pone.0004888

**Published:** 2009-03-18

**Authors:** Marie Bourjade, Alice de Boyer des Roches, Martine Hausberger

**Affiliations:** 1 Université de Rennes 1, Laboratoire d'Ethologie Animale et Humaine, Centre National de la Recherche Scientifique, Rennes, France; 2 Association Takh pour le cheval de Przewalski, Station Biologique de la Tour du Valat, Arles, France; Centre de Recherches su la Cognition Animale - Centre National de la Recherche Scientifique and Université Paul Sabatier, France

## Abstract

**Background:**

Adults play an important role in regulating the social behaviour of young individuals. However, a few pioneer studies suggest that, more than the mere presence of adults, their proportions in social groups affect the social development of young. Here, we hypothesized that aggression rates and social cohesion were correlated to adult-young ratios. Our biological model was naturally-formed groups of Przewalski horses, *Equus f. przewalskii*, varying in composition.

**Methodology/Principal Findings:**

We investigated the social interactions and spatial relationships of 12 one- and two-year-old Przewalski horses belonging to five families with adult-young ratios (AYR) ranging from 0.67 to 1.33. We found striking variations of aggression rates and spatial relationships related to the adult-young ratio: the lower this ratio, the more the young were aggressive, the more young and adults segregated and the tighter the young bonded to other young.

**Conclusion/Significance:**

This is the first study demonstrating a correlation between adult-young ratios and aggression rates and social cohesion of young individuals in a naturalistic setting. The increase of aggression and the emergence of social segregation in groups with lower proportions of adults could reflect a related decrease of the influence of adults as regulators of the behaviour of young. This social regulation has both theoretical and practical implications for understanding the modalities of the influence of adults during ontogeny and for recommending optimal settings, as for instance, for schooling or animal group management.

## Introduction

Evidence of the vast role played by human adults and adults of some animal species in regulating the social behaviour of young is now widespread [1], [2]. Experienced adults can be essential in providing models for learning behaviour socially (e.g. response of young to novel food: lambs, *Ovis aries* [3], common marmosets, *Callithrix jacchus* [4]) and above all, as models for producing and using adequate social signals efficiently [1], [5], [6]. For example, young brown-headed cowbird males, *Molothrus ater*, raised without adults were able to produce potent songs, but were unsuccessful when courting females because they failed to direct songs towards females and formed less consortships than males raised with adults [7], [8]. The absence of adults also affects language development of human children, *Homo s. sapiens*, and associates with externalizing problems in human adolescents [9], [10].

Such deleterious effects on the development of social behaviour due to the absence of adults have been widely reported in songbirds (e.g. brown-headed cowbirds [8], [11], European starlings, *Sturnus vulgaris* [12]) and, to a lesser extent, in mammals (e.g. African elephants, *Loxodonta Africana* [13], domestic horses, *Equus ferus caballus* [14]). Indeed, direct evidence shows that the lack of older elephants in a population triggers the expression of aberrant behaviour and hyper-aggression in young males that, however, can be successfully reduced by re-introducing adult males [2], [13]. Likewise, the introduction of adult domestic horses in same age - same sex groups of young appeared to decrease aggression rates and increase positive social interactions, compared to controls without similar introductions [14].

Beyond the mere presence or absence of adults, the adult-young ratio (i.e. the relative proportion of adults in a group) appears to be an important factor for social development, both in animals and in humans. Thus, this ratio plays an essential role in song acquisition in European starlings [15], [16] and in human language development [17]. A high adult-child ratio in child-care centres is one of the strongest predictors of positive care-giving and high cognitive developmental rates of children [18], [19]. Children are more responsive to their environment, have higher language acquisition rates and better global communication skills when adult-child ratios are high [20], [17]. An anecdotal report of the effects of adult-young ratios on social behaviour is also given by Campbell monkeys, *Cercopithecus c. campbelli*: the removal of two adult females from a captive group led, among others, to the increase of aggression rates between young cage-mates [21].

However, no studies have systematically investigated the potential link between adult-young ratios and other aspects of social behaviour of young animals, such as aggression rates and social cohesion, especially in natural settings. Here, we observed naturally-formed groups with various adult-young ratios and hypothesized that adult-young ratios would influence aggression rates and social cohesion of young Przewalski horses, *Equus f. przewalskii*. Young horses live in parental families (including one adult stallion, usually their father, two to five mares including their mother and their one- to three-year old offspring) until sexual maturity (two to three years) [22]. Pioneer studies showed that young domestic horses are sensitive to adult influences (e.g. mothers [23], [24], unrelated adults [14]). Here, we show for the first time that adult-young ratios are directly correlated to the expression of aggression and to the social cohesion of young horses in their family group, aspects that affect the social skills of young horses.

## Results

The 12 focal animals were one- and two-year-old Przewalski horses belonging to five families with adult-young ratios (AYR = number of horses over three years old in the group / number of horses three years old and under) ranging from 0.67 to 1.33. Aggressive and positive interactions and spatial proximity to the nearest neighbour were recorded during direct observations. Spatial relationships were then assessed by calculating the proportion of time spent with each nearest neighbour and the corresponding inter-individual distances, to estimate affinities between individuals [25, see methods].

**Figure 1:**
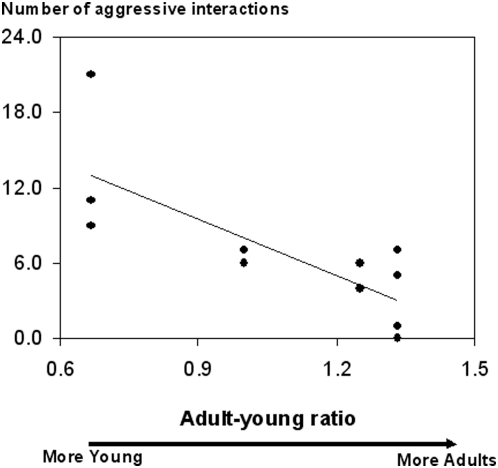
Aggression by young in relation to adult-young ratios in their family groups. *N*: number of aggressive interactions performed by young in 10 hours. Kendall partial coefficient correlation, p<0.02.

Aggression rates and spatial relationships varied significantly with adult-young ratios when the factor group size effect was kept constant (see methods). Adult-young ratios were negatively correlated with aggression rates (Kendall partial coefficient: T = −0.54, p<0.02, Fig. 1), which were up to four times higher in groups with the lowest proportions of adults (Na: mean number of aggressive interactions; Na _(AYR = 1.33)_ = 3.25±1.65; Na _(AYR = 0.67)_ = 13.67±3.71).

**Figure 2:**
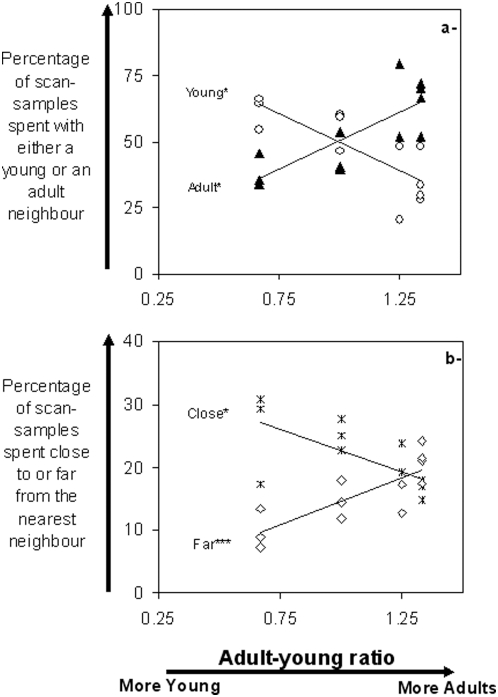
Spatial relationships of young Przewalski horses with their nearest neighbour in relation to adult-young ratios. a- Time spent with either a young neighbour or an adult neighbour, in relation to the adult-young ratio, b- Time spent close to or far from the nearest neighbour, in relation to the adult-young ratio. Time is expressed in percentage of scan-samples recorded in the field. Black triangles: adults; white circles: young; white squares: far from nearest neighbour (farther than 3.5 Horse Body-Length); stars: close to nearest neighbour (less than 0.5 Horse Body-Length). Categories “close” and “far” are not exclusive alternatives. Kendall partial coefficient correlation: * p<0.05, *** p<0.01.

Adult-young ratios were also negatively correlated to time spent close to young nearest neighbours: the higher the proportion of young, the longer young remained close to their nearest neighbours, especially young neighbours to the detriment of adults (Fig. 2ab; Kendall partial coefficients: time spent at less than 0.5 horse body-length from nearest neighbour, T = −0.44, p<0.05; time spent with a nearest young neighbour, T = −0.44, p<0.05; time spent at more than 3.5 horse body-length from nearest neighbour, T = 0.66, p<0.01; time spent with a nearest adult neighbour, T = 0.44, p<0.05).

We therefore verified that the preferences/avoidances of young for partners were not merely proportional to the availability of partners in their group (see methods for calculations). Analyses revealed that the lower the adult-young ratio, the more young avoided some partners (Kendall partial coefficient: T = −0.53, p<0.02), especially adults: young had an adult as their nearest neighbour consistently less often than expected by chance (in all groups: mean number of young partners avoided = 0.58±0.23, mean number of adult partners avoided = 2.42±0.26; one-sample permutation test: p = 0.002).

However, adult-young ratios significantly influenced neither spatial partner preference (i.e. number of preferred spatial partners, see methods), nor quantity of positive contacts, nor distribution in the other distance classes (Kendall partial coefficient: p>0.05 in all cases).

## Discussion

This study clearly demonstrates how the ratio of adults to young in social groups of horses correlates with aggression rates and social cohesion. Thus, we showed that when, in a group, adult-young ratios were low, young horses were more aggressive, segregated more from adults and established tighter bonds with other young. This is, to our knowledge, the first study investigating the effects of adult-young ratios on aggression rates and social cohesion.

The adult-young ratios observed in this Przewalski horse population are similar to those observed in feral horse populations (e.g. from 0.50 to 1.30 [22], [26]). The fact that aggression rates of young and their spatial relationships varied with this ratio means that developmental pathways experienced naturally by young can differ significantly in relation to group composition. These findings highlight the major role adults play in channelling aggressive behaviour (e.g. African elephants [6], [13], cowbirds [11], domestic horses [14]), but they also suggest strongly that the influence of adults depends on their relative numbers. This therefore raises the important question of the consequences of early-stage variations of adult-young ratios on the long-term acquisition of social skills as well as on the functioning and the efficiency of groups. Only long-term experimental manipulations of adult-young ratios in groups could fill this gap in our understanding.

In addition, we found that young in groups with lower proportions of adults formed closer relationships and more selective bonds with peers than did young in groups with higher proportions of adults, who spent more time with an adult nearest neighbour, although it was farther away. Although the presence of adults sometimes seems to induce closer associations between young (e.g. cowbirds [11], domestic horses [14]), other studies [15], [16], including this one, specify that associations between young could depend on the adult-young ratio: low proportions of adults in social groups induce more social segregation between young and adults than do high proportion of adults. These results are of interest for understanding developmental processes because social learning usually mirrors social preferences in groups [27]. Songbirds provide a good example of this process. Young starlings learn more from their preferred associates [28], and adult-young segregation, even in the presence of adults, induces young to neglect and to learn less songs from adult tutors [29], [15]. The best adult-young ratio to induce song learning from adults appears to be 1∶1 [15], [16]. Lower attention paid by young to adults induces deficiencies in their central sensory area similar to those observed in socially-deprived animals, thus social segregation and physical separation from adults can have similar effects [30].

Interestingly, a decrease of selective attention appears spontaneously in children too: toddlers pay less attention to their mothers during visits by a same-age peer [31]. Adult-child ratios in child-care centres are known to affect the cognitive and language development of young through the quantity and quality of adult-child interactions [17]. When adults are proportionally fewer in relation to the number of children, caregivers tend to interact less with the children and to use more authoritarian and restrictive speech that does not stimulate language development and learning [19], [32].

In horses, the exclusive relationship between foal-mare dyads induces foals to learn from their mothers since birth [23], but the strength of their mothers' influence decreases as the foals grow older [24]. However, one- and two-year-old horses are susceptible to the influence of unrelated adults [14]. Globally, these findings suggest that partners other than mothers could act as social models for older young, but that the proportion of young present in a group could regulate this influence by impairing either attention paid to adults or accessibility to adult partners [11], [15]. In a sense, tighter bonds between young in groups with low proportions of adults could be a factor decreasing attention paid to adults and could probably reduce their influence as regulators of the behaviour of young, in particular their aggressive behaviour. However, at this point more research is needed to explain how adult partners, considered as social stimulations of the development of young, are shared by young members of a social group and how young horses establish their social preferences.

Furthermore, this study raises fundamental questions about the impact of adults on the development of young and opens a new line of research investigating the influence of various factors implied. Thus, adult-young ratios appear to be an important feature of social settings that must be taken into account as a potential modulator of social influence when evaluating developmental processes. Furthermore, this is true for child-adult ratios in classes, which is a concern of American [17] or European legislations, but not some countries such as France (Eurydice: http://eacea.ec.europa.eu/portal/page/portal/Eurydice/).

This insight therefore has practical implications as it highlights the importance of taking adult-young ratios and their effects on learning and social behaviour into account, both for management of animal groups and potentially child schooling.

## Materials and Methods

### Focal animals and housing conditions

The studied population of Przewalski horses (*Equus ferus przewalskii*) lives in a 380 ha enclosure of highland steppe at Le Villaret on the Causse Méjean (southern France). This population grew from 11 individuals brought from zoos in 1993 and 1994. Groups then formed naturally without human intervention until 2003, when there were 55 individuals. Observations were recorded during two periods (May and June 2004; April and May 2005) and focused on five families of seven to 12 horses with adult-young ratios similar to the ratios previously observed at the study site and in their natural environment [22], [26]. We calculated adult-young ratios by dividing the number of horses that were over three years old in the group by the number of horses that were three years old and under, as Przewalski horses are adult when they are three years old [33]. Our focal animals were 12 juveniles from these five families: three one-year-old males, three one-year-old females, three two-year-old males and three two-year-old females (Table 1).

**Table 1:** Focal horses in their family groups.

Family number	Focal horse
Family 1	1f5, 1f6, 1m5 (N total = 12)
Family 2	1f7, 1m11 (N total = 9)
Family 3	1m6, 2f2, 2f3 (N total = 10)
Family 4	2f4, 2m4 (N total = 7)
Family 5	2m12, 2m7 (N total = 7)

Each horse was given an individual code indicating its age, sex and individual number. Ex: 1f5 is a one-year-old female, number five.

### Behavioural observations

We observed our subjects for two hours, twice a day, during five time-slots covering the day-light period: 0700–1000 hours, 1000–1300 hours, 1300–1530 hours, 1530–1800 hours and 1800–2100 hours. During each observation session, 10-minute scan-samples recorded the whole group, and each focal horse was observed continuously for 10-minute sampling sessions [34]. Each horse was observed for 10 hours in all. The two observers involved each year (M. B. & M. M. in 2004, M. B. & A. B.R. in 2005) recorded 25% of the observation sessions simultaneously to improve data reliability, which was controlled using Cohen's [35] kappa coefficient, that was k = 0.95.

Group scan-samples recorded the identity of, and distance to, nearest neighbour as spatial proximity is commonly used to estimate affinities between horses [25]. Distances to nearest neighbour were scored by classes of 0.5 Horse Body-Lengths (HBL) from 0.5 to 3.5 HBL and by a class (i.e. far) when distances were greater than 3.5 HBL. “Close” distances to the nearest neighbour corresponded to 0.5 HBL. “Far” distances to the nearest neighbour were scored for all distances exceeding 3.5 HBL from the nearest neighbour. Times spent in each distance class and near different neighbours were calculated in percentages of scan-samples, before analysis. Social interactions were recorded continuously during focal sampling and were expressed as number of occurrences for 10 hours. Social interactions were divided into two categories: (i) aggressive interactions, including head-threat, kick-threat, bite, kick and chase and (ii) positive contacts in the group, including approach, olfactory investigation, mutual grooming and head-body contact.

### Statistical analyses

Preferential spatial partners were identified within family groups and based on spatial proximity to nearest neighbours. Spatial preferences were not necessarily reciprocal; for instance, although individual A is B's nearest spatial partner, A's nearest neighbour could be either B (reciprocity) or C, another individual closer to it than B (non-reciprocity). Individual A's preferential spatial partners were individuals that were the closest to A more frequently than expected by chance (partitioned chi-square goodness-of-fit test, Siegel & Castellan, 1988). Avoidance of spatial partners was calculated following the same method.

As group size varied among families, Kendall partial correlation coefficients (Kendall partial coefficient) analysed our data by keeping group size constant [36]. A one-sample permutation test compared the number of young and adult partners significantly avoided by the young in all groups. All tests were two-tailed, with a significance threshold of 0.05, and performed under StatXact 4.0.1 (Cytel Software Corporation). All means are given ±SE.
